# Feasibility of implementation of CARD™ for school-based immunizations in Calgary, Alberta: a cluster trial

**DOI:** 10.1186/s12889-021-10247-4

**Published:** 2021-02-01

**Authors:** Anna Taddio, Joanne Coldham, Charlotte Logeman, C. Meghan McMurtry, Cheri Little, Tracy Samborn, Lucie M. Bucci, Noni E. MacDonald, Vibhuti Shah, Cindy Dribnenki, Joanne Snider, Derek Stephens

**Affiliations:** 1 Leslie Dan Faculty of Pharmacy, University of Toronto, 144 College Street, Toronto, Ontario M5S 3M2 Canada; 2 Alberta Health Services, 10101 Southport Road SW, Calgary, Alberta T2W 3N2 Canada; 3 The Hospital for Sick Children, 555 University Avenue, Toronto, Ontario M5G 1X8 Canada; 4 Department of Psychology, University of Guelph, 4003 Mackinnon Building (Mackinnon Extension), Guelph, Ontario N1G 2W1 Canada; 5 Cochrane Community Health Centre, Alberta Health Services, 60 Grande Blvd, Cochrane, Alberta, T4C 0S4 Canada; 6 Immunize Canada, 404-1525 Carling Avenue, Ottawa, Ontario K1Z 8R9 Canada; 7 IWK Health Centre, Dalhousie University, 5850/5980 University Avenue, Halifax, Nova Scotia B3K 6R8 Canada; 8 Mount Sinai Hospital, 600 University Avenue, Toronto, Ontario, M5G 1X5 Canada; 9 Alberta Health Services, Suite 104 Main Floor West Tower, 14310-111 Avenue, Edmonton, Alberta T5M 3Z7 Canada

**Keywords:** School immunization/vaccination, Pain management, Child, Vaccine hesitancy, Feasibility study

## Abstract

**Background:**

Negative experiences with school-based immunizations can contribute to vaccine hesitancy in youth and adulthood. We developed an evidence-based, multifaceted and customizable intervention to improve the immunization experience at school called the CARD™ (C-Comfort, A-Ask, R-Relax, D-Distract) system. We evaluated the feasibility of CARD™ implementation for school-based immunizations in Calgary, Canada.

**Methods:**

In a mixed methods study, two Community Health Centres providing immunization services, including 5 schools each with grade 9 students (aged approximately 14 years), were randomized to CARD™ or control (usual care). In the CARD™ group, public health staff and students were educated about coping strategies prior to immunization clinics. Clinics were organized to reduce fear and to support student’s choices for coping strategies. Public health staff in the CARD™ group participated in a focus group discussion afterwards. We sought a recruitment rate of 80% for eligible schools, an external stakeholder focus group (e.g., school staff) with 6 or more individuals, 85% of individual injection-related data acquisition (student and immunizer surveys), and 80% absolute agreement between raters for a subset of data that were double-coded. Across focus groups, we examined perceptions of acceptability, appropriateness, feasibility and fidelity of CARD™.

**Results:**

Nine (90%) of eligible schools participated. Of 219 students immunized, injection-related student and immunizer data forms were acquired for 195 (89.0%) and 196 (89.5%), respectively. Reliability of data collection was high. Fifteen public health and 5 school staff participated in separate focus groups. Overall, attitudes towards CARD™ were positive and compliance with individual components of CARD™ was high. Public health staff expressed skepticism regarding the value of student participation in the CARD™ system. Suggestions were made regarding processes to refine implementation.

**Conclusion:**

While most outcome criteria were satisfied and overall perceptions of implementation outcomes were positive, some important challenges and opportunities were identified. Feedback is being used to inform a large cluster trial that will evaluate the impact of CARD™ during school-based immunizations.

**Trial registration:**

The trial is registered at ClinicalTrials.gov (NCT03948633); Submitted April 24, 2019.

## Background

School-based immunizations are an efficient and accepted method of immunizing youth in many countries worldwide, including Canada [1]. Until recently, the immunization experience of students in this context was not addressed as a vaccine hesitancy concern. We now know that students experience fear, pain and other anxiety-related symptoms during vaccine injections and that having such negative experiences can undermine immunization acceptance and compliance [2]. To address this issue, we developed a person-centred framework [3] which promotes student participation and coping during immunization called the CARD™ (C-Comfort, A-Ask, R-Relax, D-Distract) System [4]. CARD™ incorporates evidence-based interventions and student preferences in the planning and delivery of immunizations in the school setting. Students are educated about CARD™ ahead of immunization day and then asked what CARDs they want to *play* on immunization day. They select from the four different letter categories - for instance, they may select the ‘Distract’ card and use their cell phone as a distraction agent.

In a controlled cluster trial conducted in Niagara, a small urban region of Ontario, Canada, CARD™ reduced high levels of fear and dizziness in grade 7 students and was so valued by the students and public health nurses that all schools in that region are now using this program [5, 6]. In the current project, we explored how to customize CARD™ before scaling the implementation. This is important given the considerable differences that may exist in: processes used to deliver immunizations among geographical regions, developmental and maturity levels of the children across grade spans targeted for immunizations at school, as well as socioeconomic and cultural factors. This study addresses tailoring CARD™ to a large urban region, Calgary, Alberta, whereby all routine vaccines are administered across the province by one health provider, Alberta Health Services (AHS), either in their public health offices or at school. This contrasts with Niagara, whereby only grade 7 immunizations are typically administered by the public health authority while earlier childhood immunizations are given in physicians’ offices. In addition, Alberta targets both grade 6 and grade 9 students for mass school immunizations and includes a diverse population, in terms of socioeconomic status and cultural background. This work was the second of a three-phase project aimed at customizing and implementing CARD™ for grade 6 and 9 school-based immunizations in Calgary. In phase one, we sought input from stakeholders to inform alterations to CARD™ resources and implementation approaches for school-based immunizations in grade 6 and in grade 9 students [7, 8]. In the current project (phase two), we trialed CARD™ implementation in grade 9 students in a small number of schools using a randomized controlled trial design. Our specific objectives were to test recruitment, data collection/study processes and implementation outcomes (acceptability, appropriateness, feasibility, fidelity). The results will inform a large randomized cluster trial evaluating the impact of CARD™, which is the final phase of the project (phase three).

## Methods

### Theoretical frameworks

The Consolidated Framework for Implementation Research (CFIR) [9] was used to guide items to probe in data collection and analysis related to implementation barriers and facilitators. CFIR is a comprehensive framework that identifies 31 constructs that positively and negatively influence implementation of complex interventions. We applied the constructs identified in CFIR to the taxonomy of implementation outcomes proposed by Proctor to describe the overall implementation success of CARD™ [10]. Four of the 8 categories of implementation success were included as intermediate outcomes of CARD™‘s treatment effectiveness: 1) acceptability: satisfaction with various aspects of the intervention (e.g., content, complexity, comfort, delivery, credibility); 2) appropriateness: perceived fit of intervention; 3) feasibility: extent to which CARD™ can be carried out; and 4) fidelity: compliance and quality of implementation. Our expectation is that the effectiveness of CARD™ will be influenced by the success of implementation. Hence, it is important to address identified implementation barriers and challenges because they provide the context for interpreting clinical effectiveness.

### Design, setting and participants

We employed quantitative and qualitative methods, including a small feasibility cluster trial and focus group interviews. AHS services are segregated into five health zones where Community Health Centers (CHCs) or service locations offer a variety of programs at the community level, including school immunization programs. The study setting included two CHCs in Calgary providing immunization services to jurisdictional schools. These two CHCs were selected from a pool of 8 primarily urban CHCs randomized to intervention (CARD™) or control (usual care) groups for phase three of the project (large cluster trial). The present study took place during the third round of grade 9 immunizations in the spring of 2019. Within both CHCs, 5 schools were randomly selected by drawing folded slips of paper with the names of schools written on them from an opaque envelope including all available schools. The principals of selected schools were subsequently invited to participate via an invitation letter.

In the Calgary school immunization program, public health school nurses are responsible for liaising with schools to organize immunization clinics, deliver education, and oversee the clinics. Immunizing nurses administer the vaccines. Managers oversee staffing, training and work processes. Administrative staff support the program, including organizing supplies and attending the clinics and assisting with workflow and supervising students.

### Study procedures

Ethical approval was obtained from the University of Toronto Health Sciences Research Ethics Board (REB) and the REBs for the School Boards of participating schools. Informed consent was obtained from all focus group participants. Consent was waived by the REB for the remainder of the study data to allow collection of population level data. Hence, parent and/or student consent was not required. Principals, however, were able to refuse participation of their school.

#### Intervention group

Table 1 provides a summary of the key phases and activities undertaken by public health staff in the intervention CHC (also reviewed in staff training video [11]). Briefly, staff involved in the immunization program were trained in the intervention in a 1-day workshop, which included presentations by study team members, including content experts and formally appointed internal implementation leaders, about: rationale for the project, scientific evidence, alignment with organizational values/mission, relevant policies and work processes. They also reviewed point of care resources (e.g., pamphlets, videos). Draft work process checklists to be used to organize and track immunization and CARD™-specific activities were incorporated into the training to allow for practice and problem-solving of potential barriers. During the training, staff provided comments which were used to inform the final implementation plan and process checklists used in the present study. All final resources were provided to staff via immediate managers, with support provided by two formally appointed local implementation leaders (TS, CL).

**Table 1:** CARD™ Framework for Immunization Delivery – Key Phases and Activities

Phase	Key Activities
Preparation of staff in the school immunization program	Educate staff about CARD™
	Tailor CARD™ processes to local context
School immunization preparation/planning	Secure adequate clinic space for immunization clinic
	Educate students about CARD™
	Students choose CARD™ strategies they want to use during immunization
	Send reminders (students, parents, school staff)
Immunization day	Minimize visual cues that elicit fear (e.g., create separate waiting spaces, obscure equipment and other students getting immunized from view)
	Introduce immunizers and review CARD™ preferences
	Identify and triage students with fear and special requests (e.g., privacy)
	Use CARD™ during immunization including inquiring about student fear and honoring student coping requests

See also related training video [11]

**Fig. 1:**
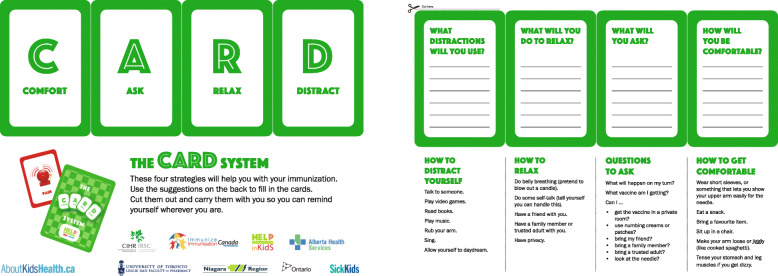
Student CARD™ Pamphle. Source: Reproduced courtesy of Anna Taddio, Professor, Leslie Dan Faculty of Pharmacy, University of Toronto, Canada

CARD™ was then integrated into school immunization program activities for included schools, including pre-immunization preparation (e.g., planning of clinic spaces, student education). Figure 1 displays a resource distributed to students by public health nurses during student education sessions prior to the immunization clinic. Students used the pamphlet to record preferred coping strategies for the upcoming clinic. On immunization day, staff set-up clinics, organized student flow and modified interactions with students according to the principles of CARD™ (Table 1). CARD™-trained staff did not communicate about the intervention with those in the control group, nor did they deliver care to schools in the control group.

#### Control group (usual care)

Public health staff in the control CHC were unaware of the intervention and followed usual practices, which excluded systematic approaches to: planning immunization clinic spaces, education of students about CARD,™ reducing cues of fear on immunization day (e.g., clinic-setup), and active student participation during immunization injections.

### Data collection

All tools and methods were adapted from our prior research with CARD™ [4]. Group-specific (CARD™ and control) versions of process checklists were completed by public health staff to guide and document their activities, including compliance with core components of CARD™. This included a student education checklist with details about CARD™ education provided to students in CARD™ schools ahead of time, and an immunization day checklist with details about what was done on immunization clinic day. During student immunizations, all nurses additionally completed a nurse feedback form after each interaction with a student, whereby details regarding individual student gender, vaccine(s) administered, and coping strategies used by students during injections were documented. Separately, all students independently completed an immunization symptom survey reporting on their level of fear, pain, and dizziness, each rated on separate 0–10 numerical rating scales. Data obtained from these checklists and forms are displayed in Tables 2, 3, 4 and 5 (see Results section of manuscript). Blank checklists and forms are available on request.

An observer independently recorded the same data as public health staff for selected process checklists and data forms (described further in Outcomes section of manuscript); this was planned for at least 2 schools (up to 4 schools) and for approximately 5–10% of students undergoing immunization, including intervention and control schools.

After clinics were undertaken in study schools, a purposive sample of public health staff implementers participated in a focus group at a public health head office. Individuals from different roles were sought to allow for a variety of perspectives, including school nurses, immunizing nurses, managers and administrative staff. The focus group discussion was facilitated by one of the study team members (AT) using a semi-structured interview guide (available upon request) and focused on CARD™ implementation, including components of CARD™ that were implemented and how they worked, confidence in ability to implement CARD™, CARD™ processes, and required resources. Public health staff rated their agreement with attitude and belief statements about CARD™ using a 5-point Likert scale (anchors: 1 = strongly disagree, 5 = strongly agree). Suggestions for phase three of the project were also recorded. Invitations for other stakeholder groups (i.e., school staff, parents, students) to participate in separate focus groups at the school were overseen by local implementation leaders, with input of the school nurses. Focus group discussions with external stakeholders and discussion notes from study team meetings were used to supplement the data and identify additional suggestions for refinement of CARD™ implementation.

### Outcomes

Below is a summary of the outcomes, level of analysis (cluster-school or individual), and associated a-priori success benchmarks.

#### Recruitment and attrition rates

- Percent of schools recruited (via principal agreement), cluster level: 80% or more
- Focus group recruitment (either school staff, parents, or grade 9 students), individual level: 6 individuals or more
- Percent of student immunization symptom surveys returned with data (yes/no), individual level: 85% or more (*primary outcome*)
- Percent of nurse feedback forms returned with data (yes/no), individual level: 85% or more

#### Implementation outcomes

- Percent of core components of CARD™ delivered (yes/no), cluster level: 80% or more
- Perceptions about CARD™, assessed via agreement with attitude statements about CARD™ (5-point Likert scale) and focus group discussion, individual level. Information was supplemented with focus groups in other stakeholder groups, as available. Suggestions for future refinement of implementation and reporting were tracked from focus group feedback and discussion notes from weekly team meetings to inform phase three of the project.

#### Reliability of data collection

- Percent agreement with 17 items included in the CARD™ compliance checklist (yes/no), cluster level: 80% or more. Data were abstracted from two process checklists containing the relevant information: student education process checklist and immunization day checklist.
- Percent agreement with 18 items included in the nurse feedback form (yes/no), individual level: 80% or more. These items track student gender, vaccination administration details, and coping strategies used.

### Sample size

The a priori sample size calculations were based on the primary outcome (i.e., percent of student immunization symptom surveys completed). A sample size of 215 (108/group) was required to obtain a value of 85% with a 2-sided 95% Confidence Interval (CI) of 80 to 90%. Clustering was not taken into account. Ten schools (5/group) were invited with the expectation that the requisite number of students would be included, with a buffer to include more students to account for fluctuation in grade 9 census (approximately 260 altogether). Focus group interviews were planned for 3–24 public health staff and managers, and 3–12 individuals in all other stakeholder groups.

### Data analysis

Quantitative analysis consisted of descriptive statistics (central tendency and variation). Qualitative analysis followed a stepwise process that included the use of two theoretical frameworks: CFIR [9] and Proctor’s implementation outcomes framework [10]. First, notes, process checklists and verbatim transcripts of focus group discussions were reviewed to obtain an overall understanding of all of the data. Then focus group transcripts were reviewed by three researchers (AT, CL, VS) and coded using directed content analysis with CFIR as the coding framework. NVivo 10 was used to organize the results. Next, the CFIR codes were reviewed by three team members (AT, CL, CMM) and subsumed within the four pre-specified overarching implementation domains (or themes) selected a-priori to describe overall implementation success: acceptability, appropriateness, feasibility and fidelity. One researcher (AT) then reviewed all of the data again and made slight modifications to the coding scheme, which were subsequently reviewed by other researchers (CMM, CL, VS) until a consensus was reached. The results of the qualitative analysis are organized by implementation outcome (i.e., acceptability, appropriateness, feasibility and fidelity), along with the relevant implementation barriers or facilitators (as defined in CFIR) identified within each of the outcomes.

## Results

**Fig. 2:**
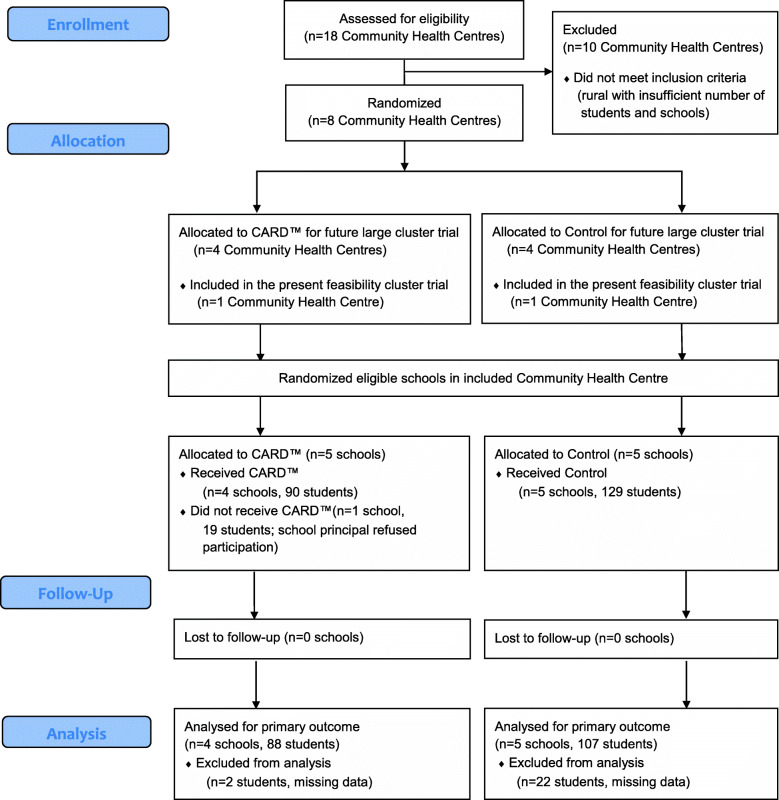
Flow Diagram

Figure 2 displays the Flow Diagram for participants in the study. The data were collected between April 25 and June 11, 2019. Altogether, 9 schools participated: 4 were in the CARD™ group and 5 were in the control group. The geographical zones of included schools revealed a similar mean number of grade 9 students in each school (103 and 110) and percentage of immigrants (33 and 33.6%) in the groups. The median household income was $115,000 Canadian dollars (CAD) and $109,000 CAD, respectively. Of 219 students recorded as immunized, 90 were in the CARD™ schools and 129 were in the control schools (Fig. 2).

Table 2 displays the characteristics of immunized students, as obtained from student immunization symptom surveys and nurse feedback forms on immunization day. Two separate focus groups were conducted: one with 15 public health staff members in the intervention CHC and one with 5 school staff members from one intervention school. Table 3 displays the characteristics of focus group participants.

**Table 2:** Characteristics of students and immunizations, student symptoms and coping strategies used

Variables	CARD™ (***n*** = 88)*	Control (***n*** = 108)*
**Characteristics**		
No. of females	40 (45.5)	50 (47.2)^a^
Mean number of injections	1.7 (0.8)	1.7 (1.2)
**Student symptoms†**		
Mean fear score	2.0 (2.1)	3.2 (3.4)^b^
Mean pain score	3 (1.9)	3.9 (2.5)^b^
Mean dizziness score	0.5 (1.1)	0.9 (1.8)^b^
**Coping strategies used‡**		
Verbal distraction	12 (14)^c^	82 (87)^b^
External distraction/object	21 (24)	38 (36)^b^
Deep breathing	8 (9)^c^	40 (37)^b^
Peer support	2 (2)	3 (3)^b^
School staff support person	2 (2)	1 (1)^b^
Nurse support person	1 (1)	4 (4)^b^
Topical anesthetic	0 (0)	0 (0)^b^
Privacy	1 (1)	0 (0)^b^
Lying down	0 (0)	1 (1)^b^
Muscle tension	2 (2)	14 (13)^b^

Values are frequency (percent) or mean (standard deviation)

* Data obtained from nurse feedback form and student symptom survey; *n* = 2 missing in CARD™ group and *n* = 21 missing in control group

† measured by student self-report on a 0–10 scale with higher values indicating higher severity

‡ measured by immunizing nurse as present/used or absent/not used during immunization

^a^ *n* = 106; ^b^ *n* = 107; ^c^ *n*=87

**Table 3:** Demographic characteristics of focus group participants

Variables	Public Health staff (***n*** = 15)	School staff (***n*** = 5)
Age, years	45.5 (11.9)	41.8 (9.5)
Sex, female	15 (100)	3 (60)
Role in organization	School Nurse: 6 (40)	Grade 9 teacher: 3 (60)
	Immunizing Nurse: 3 (20)	Vice Principal: 1 (20)
	Manager: 3 (20)	Principal: 1 (20)
	Administrative staff: 3 (20)	

Values are frequency (percent) or mean (standard deviation)

### Recruitment and attrition

Of 10 school principals approached for participation, 9 (90%) agreed. In the one school that refused participation, the principal was concerned about additional workload for staff imposed by CARD™. There were 195/219 (89.0%) individual student immunization symptom surveys and 196/219 (89.5%) nurse feedback forms returned with partial or complete data. There were some issues, however, verifying the number of students immunized (records of the number of eligible students did not match number of data forms retrieved in Control schools). This may account for the imbalance between groups in incomplete data (Fig. 2). In the only external stakeholder focus group carried out, an insufficient number of individuals (less than 6) participated. No other stakeholder groups were sought for participation due to time constraints.

### Implementation outcomes

Public health staff self-reported compliance with individual components of CARD™ was calculated to be 91.2% (Table 4). Attitude survey results revealed positive perceptions about acceptability, appropriateness, feasibility and fidelity of CARD™ implementation, as evidenced by mean Likert scores of greater than 4 (agree) out of 5 (strongly agree) for most questions (Table 5). However, scores were lower for perceptions of effectiveness of CARD™ (mean, 3.8), whether it was realistic to continue to use CARD™ (mean, 3.5), and CARD™-associated documentation workload (mean, 3.4).

**Table 4:** Compliance with delivery of individual components of CARD™ (*n* = 4 schools)

Compliance with individual component of CARD™	Number (%)*
Principal planning meeting (repeated, as relevant)	4 (100)
Teacher/staff education	n/a
Parent education	n/a
Student education – video 1 (information about immunization)^a^	3 (75)
Student education – video 2 (information about CARD™)^a^	3 (75)
Student education – CARD™ pamphlet (students self-select coping strategies for upcoming immunization)^a^	4 (100)
Student education – follow-up classroom visit and Q&A^a^	n/a
Reminders of immunization day – parents^b^	3 (75)
Reminders of immunization day – students^b^	3 (75)
Reminders of immunization day – school staff^b^	3 (75)
Preferred clinic space secured (e.g., library) for clinic^b^	4 (100)
Private room secured for clinic for students that choose privacy as a coping strategy^b^	4 (100)
Separate waiting area outside the clinic with chairs^b^	3 (75)
Public health staff introductions to classes prior to clinic^b^	4 (100)
Triage students for clinic (according to fear and choices, and minimize the number of students waiting)^b^	4 (100)
Minimize visual cues in clinic (includes separation of tables, obscuring equipment from view)^b^	4 (100)
Student symptom assessment (i.e., assessing baseline fear)	4 (100)
Student-led coping (i.e., CARD™)^c^	4 (100)
Distraction kit present in clinic (items: fidget spinner, squishy ball, mental puzzle)^b^	4 (100)
Post-clinic debrief^b^	4 (100)

* Data obtained via self-report from public health staff, whereby items denoted with ^a^ = items specifically tracked in student education checklist and those denoted by ^b^ = items specifically tracked in immunization day checklist

n/a = this activity was not applied to this phase of the project

^c^ During reliability observations, it was additionally noted that some non-fearful students were not offered cards to play during injections at one school

**Table 5:** Public health staff perceptions of CARD™ (*n* = 15)

Implementation Domain and Item(s)	Mean (SD)^a^
**Acceptability**	
I understand the individual components of the CARD system	4.3 (0.5)
I am willing to try all components of the CARD system	4.3 (0.5)
I would recommend the CARD system to reduce pain and fear during vaccinations	4.1 (0.7)
I am likely to continue to use the CARD system in the future	4.0 (0.8)
**Appropriateness**	
The CARD system is aligned with our organizational goals	4.1 (0.8)
**Feasibility**	
Management supports my daily efforts in implementing the CARD system	4.3 (0.5)
I have the support I need from other personnel to implement the CARD system	4.1 (0.6)
I believe the documentation involved in the CARD system is too time consuming	3.4 (1.1)
I think it is realistic to continue to use the CARD system in our setting	3.5 (0.9)
**Fidelity (compliance and quality of implementation)**^a^	
I am confident in my ability to use the CARD system	4.1 (0.7)
I believe the CARD system is being used in my unit	4.0 (0.8)
I believe that the CARD system improves the student experience during vaccinations	3.8 (0.8)

^a^ Data obtained from participant surveys administered during focus group interview; scores range from 1 to 5 (1 = strongly disagree, 2 = disagree, 3 = neutral, 4 = agree, 5 = strongly agree)

Qualitative feedback by public health staff and school staff is summarized below, organized by each implementation outcome domain and the specific CFIR barriers/facilitators identified within them. Selected quotations are provided in Table 6; participants are identified by group (PH = public health staff, SS = school staff) and number (01, 02, 03, etc).

**Table 6:** Selected quotes from focus group discussions involving public health (PH) staff (n = 15) and school staff (SS) (n = 5)^a^

Domain	Quote
**Acceptability:** satisfaction with various aspects of the intervention (e.g., content, complexity, comfort, delivery, credibility)	
Relative advantage/disadvantage compared to standard care	(PH12): *I really liked how there weren’t as many kids in the room, or all lined up. I think that decreased their anxiety levels too ‘cause if they’re all standing up, 30 of them in the hallway, it’s chaos out there and they kind of feed off of each other…I really, really, really liked having the chairs outside for the students to sit on. Um, like I really think that made a, that made a big difference… I think having us introduce ourselves to them is a really nice thing - just ‘cause then they can, like, familiarize what, kind of like, what to expect when they go into the room or who to expect when they go in the room… We liked the privacy theme, like we’re not all sitting like this at a table. We’re separating Tables. I think that was a good thing that I liked… I really liked the PA announcements - I think that was like really a nice way to support the students: ‘Don’t forget to play your CARD. You’re going to do awesome.’*
	(PH14): *I do like that we actually ask the kids: ‘How anxious are you?’ Before it was just a visual cue that you’re looking for: if they’re visibly nervous, that’s the only time you say, ‘Oh, this kid’s nervous!’ And you get the one out of, I don’t know how many, like the rare child that’ll come in, they’ll be talking to their friends, really nice, you know. Don’t seem nervous at all, but when it comes down to a needle, they’ll pull away. Then all of a sudden, they’re freaking out, so I like that we ask that question.*
	(SS5): *It is worthwhile, I think. Because if something goes wrong with a child at the beginning of the day, and depending on what reaction and other things happen, sometimes by the end of the day there might be more issues for us, as administrators, to deal with, right? And I’m thinking about specific children, but if somebody gets teased at the beginning and then it just compounds as the day goes on, and there are other issues not necessarily related to the vaccination process, then it becomes a discipline issue. So this is just another layer, right? So if we can make this experience as normal and as um… I don’t know, just, then it just makes the rest of the day more normal and non-eventful in a bad way. Less issues.*
	(PH13): *…but um, I did have a student mention how it made him more nervous, the introduction of the CARD system.*
Design quality and packaging (including delivery)	(PH4): *When I was going through the PowerPoint, they were completely a hundred percent not interested in that.*
	(PH2): *Four out of fourteen chose to write something (on the CARD pamphlet) and the other ones left them blank….*
	(PH4): *I think a lot of kids seem to forget... two weeks before is too far. Say if your vaccine day was on Monday and maybe if you were to somehow provide a reminder to the students, more than just the email and announcements ‘cause I don’t think a lot of kids even listen to the announcements, in my opinion.*
	(PH4): *For my school the principal sent the email to parents prior to. So, I guess that’s the onus on the parents but how many of them actually then, you know, view the videos and talk to their children about it or did they just have a question of: ‘Is this a concern for you and if it’s not…’ But it may not have, you know, reminded those kids of the options that are available to them.*
Individual stage of change	(PH2): *It went well for the two that chose to bring a support person… With the privacy, there was a Grade 9 boy, who, the mum had asked that he be done in privacy. So first two rounds, we did it in privacy. He took out his CARD, and he did not write a single thing on his CARD. So the day of, I pulled him aside down the hall and said, ‘We have done you in privacy the last two times, um is there something you would like today?’ I said, ‘I’m asking because you didn’t write that down. But it’s definitely an option.’ He’s like ‘No that’s fine.’ I’m like, ‘Ok, great!’ And he’s amazing right? And then I was like I’m gonna get a call from mom now, going ‘You didn’t do him in privacy’ right? Then we had one student who in the two previous rounds was quite dizzy afterwards and so he used the ball [i.e., muscle tension]. He’s like, ‘Oh, I think I’m okay.’ I do think that made a difference for him. I mean, who knows, maybe he would have been fine anyway, we have no way of knowing right? …he wasn’t [scared]. I don’t think it is a fear thing.*
	(PH3): *I think I’m putting more value in it now, to be honest. And not that I didn’t before. Um, but seeing the videos again and then actually having the focus group with the administrators and the teachers today, and seeing how, Okay, they’re actually really buying into this. They’re not thinking, <scoffs > ‘whatever.’ You know.*
Patient needs and preferences	(PH2): *… It’s really different, if the kid really understands that they can have a say, yeah, if they really buy in and they trust and if they’re honest, it’s a, it’s better way, right? Because you can honor their request.*
	(SS4): *Well I always talk about, even just like in assessment and testing, like the more prepared you are, the less anxious you are, right? So this is a way for them to prepare, ‘cause they’re doing it ahead of time, so it’s not, like: ‘The day is here!’ and it’s, like, scary. It’s more like, ‘OK, we’ve talked about it with the nurse, we’ve filled in our sheets, we know what to expect, it’s not a surprise.’ The more prepared I am, the less anxious…*
	(PH2): *The students didn’t realize they could advocate for themselves. So I think that that’s a strong piece, that they realize: ‘Hey, I have these options and if I’m scared, I don’t have to just sit and take the needle. I have a…’ so it kind of gives an option for that. So, I like that piece.*
**Appropriateness:** perceived fit of intervention	
External policy and incentives	(PH3): *I really liked how (teacher name) was really wanting to implement it in health class and…kind of have the conversation with the kids just on any random things in their health class or whatever. Talking about, you know, using your CARDs, what CARDs would you use for that, and things like that and using the same language… Teaching the kids how to advocate for themselves and how to deal with these adverse situations, how to learn to build the resilience*… *It sort of elevates us, and it elevates our importance in the school.*
	(PH4): *I’m there to talk about health promotion and healthy eating, active living, and on and on and on. I can talk about vaccines if they want, but that’s not really something that they would approach me about. But having this, um, insight, I kind of feel a little stupid, didn’t think about this before, right? But it’s just pointing it out that these conversations need to be had, it needs to be more of a team approach for vaccination. It’s not them* versus *us.*
Reflecting and evaluating	(PH3): *Sometimes it feels like there’s the vaccinating team and then there’s the school nurse team, right? And I don’t like that division. I hate that division… At three o’clock or whenever the meetings are, (the vaccinating nurses) can come to the beginning of that meeting ‘cause then we’re more of a team… I don’t think it’s wasting time. You’re putting value to the teamwork.*
	(PH2): *I do think that there’ll be more students probably who fill (the CARD pamphlet) in in the fall. I think because most of these students have already received multiple vaccines this year at school, that this is another visit - some it was their first - but the majority of them have seen us already several times. Um, I do think if we start with it, I think it will, um, it will be good.*
Perceived Compatibility	(PH13): *I think that (injecting 2 vaccines in 1 arm) will cause more stress to some of the students*. (PH12): *I think it’ll be a little bit awkward.* (PH14): *I have to consider the space too, yeah, if the room even allows us moving around the student like that.*
	(SS5): *the coordination, if we take students out from individual classes who have a heightened level of anxiety, that’s just a little bit more prep work... In terms of the planning, we just have to be a little bit more mindful. As to the vaccine day, the times and then if we have the information about the kids then that adds another layer but it’s… it’s doable. It’s just a lot of front-end planning.*
	(SS3): *We’re talking about the anxiety of the needle. What about the anxiety of – ‘I just left math class. There’s an important lesson going on. I’m sitting on this cold bench, waiting. And I’m anxious ‘cause I’m supposed to be in that class learning this concept, and when I go* ba*ck, I’m behind’… I’d use language with your nurses: ‘Can you work with the admin team for an adapted schedule?’ Yeah - ‘Is it possible to schedule extended homeroom time? Is it possible to extend the health class?’ Ones that are typically with a comfortable teacher that they know well, and it’s not a huge disruption to an academic course where that child that’s anxious for the needle is probably very anxious for missing the big concept.*
	(SS1): *I look at this CARD system and I feel like we’ve been doing this already in this school. Um, we talk to students a lot. We send an email to parents and let them bring an extra snack and juice box, something, a phone, a stuffy, like whatever they want to bring. I feel like we communicated that and let that happen already. This is just, more I think, gonna be really for making sure that they are actually truly aware that ‘I put this in writing, so I really can, and not just my teacher said I can, but it’s in writing.’ So I think it’s just formalizing what we’re already doing in here from the most part.*
	(PH3): *My biggest worry is actually not so much the CARD thing, it’s just the individual [computer] data entry that I find is slowing things down… ‘cause it’s taking a lot more time for students… sitting there a lot longer.*
**Feasibility:** extent to which CARD™ can be carried out	
Actual Compatibility	(PH13): *I didn’t find it, um, very different. I was just adding a couple extra questions. I already…from like the task list, I already did everything on there. I was just adding a couple of extra questions, which I didn’t think made much of a difference to me on immunizing.*
	(SS3): *I sat out on the bench with all the students that you have there, there was no one formally in the room to help that anxiety that a few students were exuding. They were on that bench, and that anxiety’s now starting to spread.*
Design quality and packaging	(PH4): *The distraction items - they had no interest in even looking at them. So I feel like it wasn’t quite appropriate for their age category.*
Complexity	(PH16): *We vaccinated in the library and I really noticed that this is a challenging spot for interruptions. We almost needed to have someone stationed at the door because even though there were two signs on each door and there was an announcement that the library was closed, there were still teachers and students walking in.*
	(PH16): *And I think this school, they didn’t want them with their phones… We could not have chairs in the hallway either, that hallway is narrower than most and it would have been very difficult with class changes and lockers on both sides.*
Adaptability	(PH4): *We couldn’t do our introductions in the classroom, just because of how the Grade 9 cohort works. Um, so we did our introductions in the hall…*
	(PH2): *I did staple their CARD to their consent forms because it was my way of identifying who had chosen something* versus *who had left their CARD pamphlet blank... So it also helped me to say: ‘Do you have a support person? Would you like to go get your support person?’ prior to them going into the vaccination area. So that was my trigger to ask them.*
	(PH18): *You did the debriefing at the school but I found many ideas came at your meetings, you know, after, so that’s a way to keep it, I think, going too because you generally have the weekly meetings.*
Available resources	(PH16): *It is really a lot more staff required though. We did 37 grade 9 in about an hour and a half with 5 vaccinators. Then we did about 35 others in about 25 min.*
Reflecting and evaluating	(PH12): *We were never told what was written on the CARDs, if they were filled out. Nothing was told to us. So it was kind of blind for us. We don’t know what they’re expecting.*
	(PH3): *Time crunch, and so you’re quickly going through the debrief… it was a little bit short today. I don’t know if we’re maybe focusing on the debriefing as much as we could… Um, maybe we’re not taking it seriously enough, to be honest. Maybe [just ask]: ‘How can we do this? How can we work together as a team? How can we communicate with each other to make this the best experience that we can?’*
**Fidelity:** compliance and quality of implementation	
Executing	(PH2): *The students filled out CARD, but they almost seemed to fill it out almost like they thought it was like a test. And so I thought that was interesting. It proved that they were listening, which is fantastic, but I’m like, that’s really not the purpose of it right? [The next time] after I showed the videos, I handed out the CARD pamphlet, I had all the students put their name on it, and then said: ‘Now think about what you saw, think about what you need, if you think that there’s something you need please put it down. But it’s okay if you don’t, like if you want to leave your CARD blank because nothing resonated to you or you don’t think you need this, that’s okay too.’*
	(PH3): *I couldn’t get the videos to work [school name]… We talked about all sorts of things and we did show the PowerPoint. We just didn’t show the videos, but they still didn’t seem as prepared [as other school].*
	(PH14): *They didn’t really seem interested, I found. Like most of them, they didn’t buy in… As soon as I would ask, ‘Where are you on the fear scale?’ They would say, ‘Around a two or a one.’ And I would say, ‘What CARD do you want to play?’ And he was like, ‘I don’t want to play a CARD, I’m fine.’*
	(PH4): *There was a boy who I know sort of personally, I know his mother… So third time around now, post-CARD, he came in and I asked him what his fear level was, he said he was “two” (out of three) and I said, ‘Okay, well you know, last time this was what we did and you’ve been given some information about things that you can do. Um, so what CARD are you gonna play? What are you gonna do?’ He said, ‘Well, I got my phone.’ Okay, and he pulls out his phone, and he completely ignores me for the rest of the whole appointment, which is good for him, I mean, I’d already done my bit to immunize. And I was like, ‘Okay, d’you just want me to-“Just do it!’ And so there was no anxiety, there was no jitteriness, there was no, you know, moving around. It was just, didn’t have to worry about spelling anything, he was distracted with his phone. He had it there.*
	(PH3): *Due to time constraints today and just being they needed to get some Grade 9’s out of there because they were going (to an event). So, for ease of everything – disruption - we just called all eleven kids down because they were from three different classes. That was a little bit much for the two vaccinators. So, you know, calling four down at a time, or just like you say, double the amount of vaccinators, I think just reinforcing that.*
	(PH14): *For anxious ones, I always explain to them: You know sometimes watching it makes you more anxious, so maybe you can try looking away, maybe that will help you… at the end of it, if they’re gonna look, they’re gonna look.*

^a^ Results are organized by implementation outcome (acceptability, appropriateness, feasibility and fidelity, as defined by Proctor et al. [10]) and specific implementation barrier or facilitator construct (as defined in the Consolidated Framework for Implementation Research [9])

#### Acceptability

Four CFIR constructs were identified related to positive and negative perceptions of acceptability of CARD™; 1) relative advantage compared to usual care; 2) design quality and packaging and delivery (i.e., how the intervention is bundled, presented and assembled); 3) individual stage of change (i.e., phase of progression in the use of the intervention); and 4) identification and priority of patient needs and resources. Nurses and school staff reported that many components of CARD™ were advantageous when compared to usual practices, including: having fewer waiting students, use of a separate waiting area for students with chairs, performing team introductions to classes prior to clinic commencement, enhancing privacy in the clinic, providing school announcements reminding students of immunization day, assessing baseline levels of fear in students, and reducing the potential for discipline issues resulting from negative immunization experiences.

Nurses perceived that student and parent education may have been inadequate and believed this was due to the materials used and/or their delivery method, including timing relative to immunizations. In addition, nurses did not consistently attribute positive immunization experiences with student self-selected coping choices. For example, an immunizing nurse reported that one student was more nervous with CARD™. One nurse commented on increasing her own buy-in over time that was influenced by re-review of CARD™ educational tools and positive feedback received from school staff. School staff reported the benefits of educating students ahead of time and the empowerment of students in the immunization process.

#### Appropriateness

Three CFIR constructs related to perceptions of appropriateness: 1) external policy and incentives that spread the intervention; 2) reflecting and evaluating on the process of implementation; and 3) compatibility or degree of fit. Nurses reported that CARD™ was aligned with current activities undertaken by school nurses and that it helped to strengthen relationships with schools. Encouraging involvement of immunizing nurses in regular CHC staff meetings was recommended for building and strengthening relationships among staff. It was suggested that student education might be more effective in the fall before they experience grade 9 school immunization clinics.

Hesitancy was expressed by immunizing nurses regarding a change to practice announced by a senior manager to inject 2 different vaccines in the same arm (rather than separate arms), despite rationale for safety and efficiency provided by the manager. School administrators reported a willingness to work with public health staff to plan and accommodate requests.

One participant identified a new point-of-care immunization data entry program being implemented concurrently that increased the amount of time required for each immunization encounter, which was counter to some CARD™ measures intended to reduce time.

#### Feasibility

Six CFIR constructs related to perceptions of feasibility: 1) compatibility; 2) design quality and packaging; 3) complexity of the intervention; 4) adaptability of the intervention; 5) available resources to implement the intervention; and 6) reflecting and evaluating on the process of implementation. With respect to compatibility, immunizing nurses reported that the interaction between immunizing nurses and students fit into usual workflows. One vice principal, however, mentioned sitting with unsupervised students waiting to be immunized to assist with anxiety. Several nurses mentioned that items included in the distraction kit (squishy ball, fidget spinner, word puzzle) were inappropriate for grade 9 students. Nurses and managers commented on difficulty implementing some interventions related to school factors, including physical layout and behavior of students and staff. Nurses reported making adaptations to prescribed implementation processes with the intention of trying to achieve the underlying goal, such as; introducing themselves to students after taking them out of class rather than before and attaching completed student CARD™ pamphlets to immunization consent forms to prompt nurses about student choices. Nurses and managers identified regular staff meetings as a good venue to debrief about CARD™ implementation to inform reflections and additional refinements. One of the managers stated that additional resources were required to implement CARD™. Nurses reflected on their experiences with specific implementation processes and offered ways to improve them.

#### Fidelity

One CFIR construct related to intervention fidelity: 1) executing (carrying out or accomplishing the implementation according to plan). Nurses reported variations in compliance and quality of implementation of different aspects of CARD™. For instance, one nurse described learning to educate students more effectively after observing that students filled out their CARD™ pamphlet as if it was ‘a test’ rather than choosing their own coping strategies. Sometimes, interventions were not executed, and nurses suggested that poor fidelity with implementation may have impacted effectiveness.

Nurses reported that some students did not appear to be interested in CARD™ education and that students did not consistently choose to play any cards during immunization. Nurses also reported using usual coaching approaches during immunization injections whereby they directed students rather than engaging them in making their own choices as recommended by CARD™.

##### Suggestions for future cluster trial

Suggestions regarding additional refinement for the cluster trial are summarized in Table 7. Briefly, they include activities overseen by implementation leaders to foster a positive implementation climate, booster training for staff, additional ‘how to’ resources for operationalizing different components of CARD™ and data collection procedures, and minor adjustments to the student education.

**Table 7:** Suggestions for CARD™ refinement for future cluster trial

Aspect of implementation	Specific suggestions regarding preparation of staff for future cluster trial
Leadership roles	Foster a positive implementation climate by undertaking the following activities: • Frequent communication between formally appointed implementation leaders and project leaders regarding progress and issues related to implementation
	• Checklist for vaccine coordinator (manager overseeing school immunization program at CHC level) articulating supervisory role in staff training and CARD™ implementation
	• Process for regular data transfer (e.g., weekly) to researchers for ongoing data entry and feedback to support ongoing debriefs and planning future clinics
	• Ensure adequate staff to carry out CARD™ (plan for activities to take longer due to learning curve and additional time involved in immunization planning activities)
	• Manage staff anxiety from CARD™ and concurrent practice changes (e.g., direct data entry)
	• Regular communication (newsletter, infographic, Q&A) regarding study progress and results to engage and inform staff
	• Solicit structured feedback from external stakeholders (e.g., school staff)
	• Prepare and oversee additional training and resources with project leaders to support staff with CARD™ implementation, *as specified further below*
Booster training^a^ for staff	Provide additional training and support (overseen by the vaccine coordinator and supported by the formally appointed implementation leaders) to improve staff understanding, buy-in and self-efficacy, including:
	• Rationale and overarching goal of CARD™ and alignment with organizational mission/values
	• Re-review of educational materials (e.g., training videos)
	• Revised and expanded resources for use (e.g., invitations to principals for participation, scripts for interactions with students, class introductions, reminder prompts on data forms)
	• Detailed instructions regarding data collection procedures (e.g., use of pre-numbered data collection forms, and other checklists)
	• Role of staff feedback to inform revisions and refinement to implementation processes and documentation, *as specified further below*
Specific instructions in implementation processes and checklists	Provide more instruction (including sample pictures/illustrations) regarding how to tailor some components of CARD™ to the local context, including:
	• Bundling needed materials and supplies (e.g., table posters, door signs, distraction kits, extra data forms) ahead of time, in a *grab and go* bag, for easy retrieval
	• Leveraging existing meetings with school staff to include information about CARD™ (e.g., annual anaphylaxis training)
	• Keeping the number of students in pre-immunization waiting area to a reasonable amount (about double the number of immunizing nurses)
	• Addressing student anxiety in the pre-immunization waiting area. Suggestions: distraction kits, adult supervision
	• Providing seating in pre-immunization waiting area to increase student comfort
	• Adding more instruction about effective separation of workstations, obscuring equipment from view, and positioning students so that they cannot see one another during immunization
	• Diversifying distraction items in the distraction toolkit so they are suitable to different students
	• Including information about injecting two vaccines in the same arm
	• Using regular staff meetings to continue conversations about CARD™ implementation with all team members go inform additional revisions and refinement to implementation
Revision of data collection forms	Revise the nurse feedback form to maintain fidelity and accuracy in documentation, including:
	• Adding reminder prompts for several core components of CARD™ performed at the time of immunization (i.e., asking about fear prior to injection, inviting students to play their CARDs, injecting 2 vaccines in one arm)
	• Clarifying operational definitions of coping strategies (e.g., verbal distraction, deep breathing, and muscle tension) to reduce potential error and bias in their interpretation by immunizing staff recording data
Revision of student education	Revise student education, including:
	• Instructing students that 2 vaccines will be injected in the same arm unless they choose otherwise
	• Adding pictures of the immunizing space and waiting area that will be used in the specific school to help prepare them
	• Sending reminder to students about immunization day close to the actual date to prevent them forgetting
	• Ensuring educational videos play ahead of time (e.g., download videos onto computer, obtain necessary computer adapters, IT support)
	• Reducing teaching time around the videos, including removal of cases, to reduce redundancy/overlap with videos; use time to answer student questions
	• Encouraging students to bring their own distraction items to ensure they are suitable
	• Including a topical anesthetic information form
	• Pinning up CARD™ posters around school (e.g., main area, classroom) as a reminder of CARD™
	• Providing access to students of their completed CARD™ pamphlets so that they can be reviewed prior to immunization

^a^ Additional training in those that already underwent training to review and refresh knowledge, attitudes and skills related to implementation of the intervention

### Reliability of data collection

Overall percent agreement for compliance among 17 of 20 individual components of CARD™ included in the study was 100%. Altogether, percent agreement for 15 nurse feedback forms (6.8% of all immunized students) was 92.6% for the 18 targeted variables on the form. Discrepancies above > 20% were noted for certain coping strategies, including verbal distraction, external distraction and deep breathing. It was noted by observers at one school that some non-fearful students were not offered cards to play during injections.

## Discussion

We evaluated study procedures and implementation outcomes for a novel evidence-based framework for delivering vaccines at school in preparation for a future cluster trial. This CARD™ system framework integrates specific activities into usual school immunization planning and administration procedures that together promote person-centred care and coping. Overall, school recruitment was high as were retrieval of both student and nurse-reported outcome data. However, one principal in an intervention school declined participation due to concerns about workload. In addition, there was some confusion regarding the total number of immunized students because of a lack of accounting of all data forms. Self-reported compliance with the main components of CARD™ and reliability of data collection were high. There were some parts of the nurse feedback form, however, related to specific coping interventions, that were not consistently recorded between immunizing nurses and observers. Overall, feedback from participants was positive regarding perceptions of acceptability, appropriateness, feasibility and fidelity. However, there was skepticism regarding the value of components of CARD™ that involved students, including education about CARD™ ahead of time and student-led coping strategies during immunization injections. In addition, nurses reported challenges carrying out some aspects of CARD™ and anticipated future challenges, some of which related to limits imposed by school physical layout and routines. Limited feedback obtained from school staff revealed support for CARD™ and willingness to collaborate with public health staff in its implementation. Suggestions were made to refine future implementation and study processes.

These results are consistent with prior research demonstrating that CARD™-informed alterations in the school immunization delivery process are possible and can have positive effects on public health staff perceptions of clinic success [5, 6]. In particular, staff reported satisfaction with changes in the physical set-up and flow of students to reduce cues of fear. In addition, CARD™ was perceived as promoting interactions and collaboration between individuals in different roles within public health (school nurses and immunizing nurses) as well as across organizations (public health staff and school staff). These interactions were viewed as feasible within current work processes. Importantly, school staff were supportive of CARD™ and willing to work with public health staff to plan immunization clinics that followed the principles of CARD™.

With respect to implementation fidelity, some components of CARD™ were inconsistently applied such as education of students and offering non-fearful students cards to play during vaccine injections. Staff also reported some logistical and physical challenges related to workflow and school factors (e.g., physical layout and routines). This led to adaptations to locally prescribed implementation approaches or omitting specific activities altogether. CARD™ encompasses numerous process components, many of which were new to staff; therefore, staff may not have grasped if and how the locally prescribed implementation approaches could be adapted and to what extent. Additional training and support are recommended to provide staff with the rationale for inclusion of all components of CARD™ and options for how to implement them that consider the likelihood of achieving desired outcomes. Including staff in planning and trialing new approaches, particularly for situations whereby the optimal course of action is unclear, may also increase engagement, collaboration, evaluation and self-efficacy. This, in turn, can further enhance implementation success.

In general, nurses were skeptical about including students as active partners in the process. They reported that some students did not receive, understand or appreciate CARD™ education, and did not come prepared to *play their cards* during the immunization clinic. At the same time, when students did *play their cards*, nurses expressed uneasiness about having students leading their own coping strategies, particularly if they conflicted with the approaches that the nurses or parents preferred. This may have contributed to less positive attitudes regarding the effectiveness of CARD™ and how realistic it was to continue to use it. Engaging students as active partners during immunization and coaching them on their preferred coping strategies may represent a significant change in perspective for many nurses who are accustomed to leading interactions with students and directing them to use nurse-selected coping strategies. This finding contrasts with the experience in Niagara, whereby student involvement was perceived as a major benefit by both nurses and students [5, 6]. This finding represents an opportunity for further evaluation of staff perceptions of what is intended by a person-centred approach to immunization delivery as well as student perceptions of being included [3]. Importantly, students in the present study, unlike in Niagara, would have had prior experiences with immunizations at school, including in grade 9, which may have shaped how they reacted to the nurses and the new program during the education and then on immunization day.

In addition, nurses seemed uncertain about the role of CARD™ in fearful vs. non-fearful students. Specifically, it was unclear to them whether students who were not fearful should *play* their cards. A significant part of CARD™ addresses student coping with injection-related fear and pain; however, it is a more comprehensive framework that incorporates students as active participants in the immunization process, addressing their concerns and giving them choices and control over how immunizations are delivered in order to meet their needs and preferences. There are no “cut off” fear scores as this is a universal intervention designed to be relevant for all students and is consistent with currently espoused models of health care delivery that stress participation of individuals. Given the limited number of interactions nurses had with students who self-selected coping strategies and the late introduction of CARD™ relative to school immunization clinics in the current academic year, nurses may feel more comfortable with student-led participation with more experience and if the timing of CARD™ implementation coincides with the first immunization clinic in the academic year. To assist with this transition, additional resources and support reinforcing the rationale and assisting with practice are recommended to facilitate learning and lead to more positive attitudes about this approach. Our prior work in grade 9 students leading up to this study suggested acceptability of CARD™ for this age group [7]. As grade 9 students in the present study were not consulted about their perceptions of CARD™, additional research is recommended to find out more about how to customize CARD™ to meet their needs and preferences.

Reliability testing revealed some discrepancies in the frequency of reporting of specific coping strategies between raters, suggesting inconsistent operational definitions were being used when recording the data. With respect to verbal and external distraction, it was determined that the timeframe for the use of the strategy largely contributed to differences in ratings (i.e., whether undertaken before or during the injection). With respect to deep breathing, two separate interventions were identified that use the same name: 1) one deep breath taken just before injection, and 2) several deep belly breaths taken before and during injection. Based on these findings, there is potential for random error in this variable in the cluster trial. There is also potential for bias because of systematic differences between groups in staff training about coping strategies. Those in the CARD™ group receive more training and this may lead to systematic differences in how they recorded the use of various coping strategies. As there was no reliability data collected in control schools, the potential for bias could not be explored. The pattern of use of coping interventions, while not formally analyzed between groups in the present study, may be suggestive of bias in the reporting of some strategies (see Table 2). Potential biases may lead to a divergence in the results between groups in utilization patterns for different coping interventions and student symptoms. Ways to mitigate both error and bias are highly recommended for the cluster trial.

This study included a limited number of public health staff implementers, schools and students and not all parts of CARD™ were included. In addition, few students requested any coping interventions. This may have prevented us from identifying all potential challenges with implementation processes in preparation for phase three of the project. Future challenges may include: 1) ensuring staff educate all students due to the vast number of schools and students; 2) fidelity with carrying out certain activities because of perceived need, resource or logistical challenges; and 3) role of school staff in implementation success, including cooperation and collaboration with requested activities. Frequent communication between implementing staff and local study leaders and regular study communication (newsletter, Q&A) are recommended to identify and address arising implementation issues.

There are numerous strengths of the study related to methodologic rigor, including: randomization of schools, blinding of key individuals, inclusion of all immunization clinic-related data (i.e., all immunized students), pragmatic implementation, use of theoretical frameworks to guide data collection and interpretation, and inclusion of multiple data sources. In addition, the research team included health care providers from across disciplines and roles with experience with school immunizations. Together, these factors contributed to a more comprehensive inquiry into the implementation context for CARD™ in Calgary. A number of recommendations were made that relate to staff training and study procedures for future implementation efforts. With respect to training, additional ‘how to’ tips were recommended that identify different ways to operationalize core components of CARD™ with careful consideration of compatibility with practice. This might also include information about the associated likelihood of achieving intended outcomes with each of the options. With respect to data collection, procedures that allow for accounting of all data forms were recommended. A checklist for managers was developed to assist with overseeing of training and study procedures.

## Conclusion

This study tested recruitment procedures, data collection and study processes and implementation outcomes (acceptability, appropriateness, feasibility, fidelity) for a future cluster trial evaluating a new framework for vaccine delivery at school called the CARD™ system. Overall, study processes were successful, and participants had positive perceptions about CARD™. The findings support the ability to carry out a large cluster trial that will evaluate the effectiveness, fidelity and cost of CARD™ implementation in Calgary. Improving the experience of students undergoing immunization has the potential to reduce vaccine hesitancy, which will, in turn, improve vaccine uptake. This work is particularly relevant now, as surveys suggest that vaccine hesitancy has been steadily increasing during the pandemic, and may threaten the success of immunization programs. Additional research is therefore also recommended to adapt CARD™ for adult COVID-19 immunization.

## Data Availability

The datasets generated for this manuscript are not publicly available. Permission was obtained by the overseeing ethics board, Alberta Health Services and participating schools to collect the data; however, permission was not granted to make the datasets public.

## Abbreviations

AHS: Alberta Health Services
CAD: Canadian Dollar
CFIR: Consolidated Framework for Implementation Research
CHC: Community Health Centre
CI: Confidence Interval
